# Integrating biomarkers and artificial intelligence for precision diagnostics in cattle health and herd management: a review

**DOI:** 10.1007/s11259-026-11268-3

**Published:** 2026-05-14

**Authors:** Şeyma Aydın, Selçuk Özdemir

**Affiliations:** Department of Genetics, Faculty of Veterinary Medicine, Atatürk University, Erzurum, Türkiye

**Keywords:** Biomarkers, Cattle diseases, Artificial intelligence, Multi-omics, Precision livestock health

## Abstract

Early and accurate diagnosis of major production diseases in cattle, many of which may be subclinical, such as mastitis, ketosis, and bovine respiratory disease (BRD), is essential for herd efficiency, animal welfare, and long-term sustainability. Conventional diagnostic approaches based on clinical observation and microbiological assays often lack sensitivity for early-stage detection and show limited predictive value in multifactorial conditions, thereby limiting early risk stratification and timely intervention. Biomarkers derived from accessible matrices such as blood and milk provide valuable insight into inflammatory, metabolic, and reproductive disturbances, especially at subclinical stages. However, their clinical implementation remains limited by pre-analytical and analytical variability (e.g., sample collection, storage, and processing), the absence of standardized thresholds, and the lack of reliable cow-specific decision cut-offs. The integration of multi-omics data, including genomic, transcriptomic, proteomic, and metabolomic layers, with artificial intelligence (AI) enables high-dimensional data integration, automated classification, and predictive modeling in cattle health. While AI-driven approaches show promise in supporting biomarker network interpretation, their translation from predictive modeling frameworks to clinically validated diagnostic systems remains limited. This review critically synthesizes current evidence on the utility and limitations of biomarkers and AI-assisted diagnostics in cattle health management, while identifying key methodological constraints and outlining practical pathways toward standardized and interpretable AI-driven systems.

## Introduction

Global population growth, urbanization, and rising incomes are projected to substantially increase demand for animal-source foods by 2050, with estimates suggesting up to a 70% rise in overall demand and particularly pronounced growth in meat and milk consumption in developing regions (Turk 2016). Dairy and beef cattle are at the heart of meeting this demand, supplying the bulk of the world’s milk and meat (FAOSTAT 2024; Van Eenennaam 2024). However, the efficiency and sustainability of these production systems are highly dependent on animal health, as livestock diseases collectively impose substantial economic and welfare burdens by increasing treatment costs, extending recovery periods, elevating culling rates, impairing reproductive performance, and ultimately affecting global food security (Kappes et al. 2023).

Simultaneously, despite the rising global demand for animal-source products, the number of livestock farmers has been declining in several regions, including the European Union and the United States (Eurostat 2020; USDA 2024). This demographic shift has resulted in larger herd sizes and more complex production systems, further complicating timely and accurate disease detection (Clay et al. 2020). Consequently, the combination of growing production demands, fewer farmers, and expanding herd sizes has intensified the need for rapid and reliable biomarker-based diagnostic approaches to support effective herd health management.

**Fig. 1:**
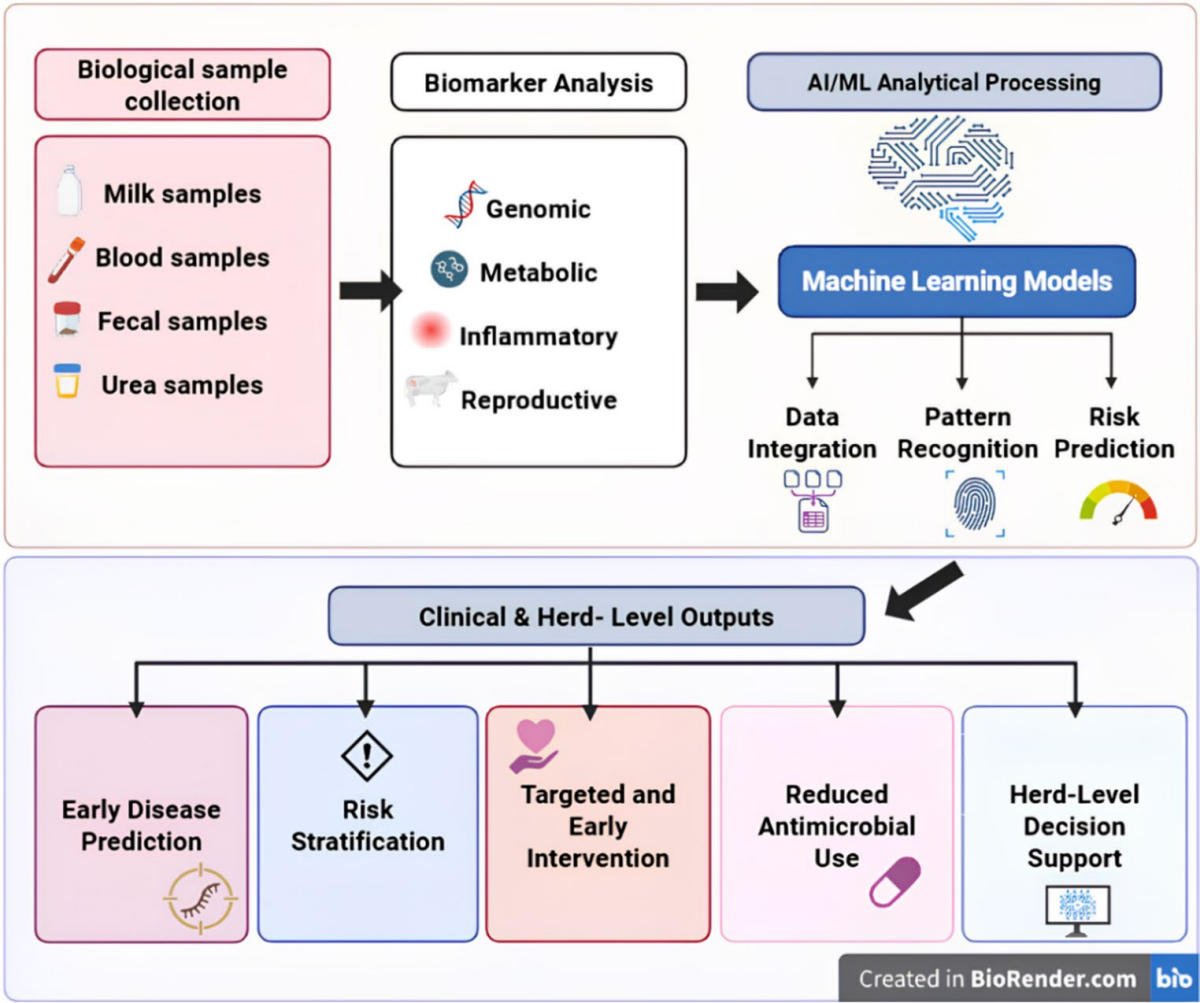
Integrated biomarker– artificial intelligence (AI) workflow for disease prediction and herd-level decision support in cattle health management. The schematic illustrates the translational pipeline linking biological sample collection (milk, blood, fecal, and urea samples) to multi-category biomarker analysis (genomic, metabolic, inflammatory, and reproductive markers), followed by AI/ML-based analytical processing. Machine-learning (ML) models perform data integration, pattern recognition, and probabilistic risk prediction to generate clinically actionable outputs. These outputs include early disease prediction, risk stratification, targeted interventions, reduced antimicrobial use, and herd-level decision support. The framework highlights the transition from threshold-based biomarker interpretation toward context-aware, data-driven disease modeling that supports precision veterinary management and sustainable livestock production

In this context, timely identification of subclinical or emerging disorders enables rapid intervention, minimizes productivity losses, and reduces reliance on broad-spectrum antimicrobials. Biomarkers are increasingly recognized as key indicators of physiological and pathological states, supporting early disease detection and herd-level health monitoring. Yet their diagnostic utility is constrained by biological variability and the complexity of multifactorial disease interactions (Perera et al. 2022). Recent advances in artificial intelligence (AI) and machine learning (ML) provide transformative opportunities to overcome these limitations. By enabling real-time, high-dimensional analysis of complex biomarker datasets, AI-driven systems may facilitate a shift from reactive to predictive herd health management. This transition could provide veterinarians with actionable insights to improve animal welfare, productivity, and sustainability (Fig. 1).

The unique contribution of this review lies in its integrative approach, bringing together the diverse biomarker categories used in cattle disease diagnostics and the AI/ML models designed to interpret these complex datasets. While most existing studies examine these two domains separately, the link between the diagnostic potential of biological markers and the high-dimensional analytical capabilities of AI has not been sufficiently articulated in the literature. By evaluating both current biomarker groups and AI-based prediction models through a comparative and integrative framework, this review aims to fill the existing gap related to early diagnosis, multi-source data integration, and field-level applicability.

## Review methodology

This narrative review aimed to synthesize current evidence on biomarkers and AI-based approaches for precision diagnostics and herd health management in cattle. A targeted literature search was conducted in PubMed, Web of Science, and Scopus using combinations of terms related to biomarkers (“biomarker,” “omics,” “genomic markers,” “metabolic biomarkers,” “inflammatory biomarkers,” “reproductive biomarkers,” “hormonal biomarkers,” “non-coding RNA,” “miRNA,” “cell-free DNA”), artificial intelligence (“artificial intelligence,” “machine learning,” “deep learning”), and cattle health (“cattle,” “dairy cattle,” “beef cattle,” “livestock health,” “herd management”).

The search was limited to peer-reviewed articles published in English between January 2015 and March 2025 to capture contemporary advances in biomarker discovery and AI-driven analytics. Grey literature (conference abstracts, preprints, technical reports) was excluded to ensure methodological rigor and reproducibility.

Approximately 850 records were identified. After duplicate removal and title/abstract screening, 120–150 articles underwent full-text evaluation, and 65 studies met the inclusion criteria.

Eligible studies included original research and high-quality reviews addressing biomarker discovery, AI-assisted diagnostic modeling, or their application in cattle health management. Given the limited number of fully integrated biomarker–AI investigations, studies focusing exclusively on biomarkers or AI-based herd prediction systems (e.g., sensor-derived behavioral data) were also included to highlight their independent strengths and potential for future integration.

Due to heterogeneity in study designs, biomarker classes, and AI methodologies, a narrative synthesis approach was employed. The conceptual inclusion and exclusion framework guiding study selection is summarized in Table 1.

**Table 1:** Conceptual inclusion and exclusion framework applied in this narrative review

Category	Inclusion criteria	Exclusion criteria
Population	Dairy or beef cattle	Non-bovine species
Biomarker Studies	Genomic, transcriptomic, proteomic, metabolomic, inflammatory, metabolic, non-coding RNA, or cfDNA biomarkers relevant to cattle health	Studies without measurable biological markers
AI-Based Studies	ML, deep learning, or AI-based predictive/classification models applied to cattle health	Studies without computational or analytical modeling
Integrated Approaches	Studies combining biomarkers and AI frameworks	Conceptual discussions without biological or analytical components
Study Type	Original research and high-quality reviews	Conference abstracts, preprints, grey literature
Time & Language	English-language articles published between 2015–2025	Non-English publications or pre-2015 studies

## Disease-associated productivity losses in dairy and beef systems

**Fig. 2:**
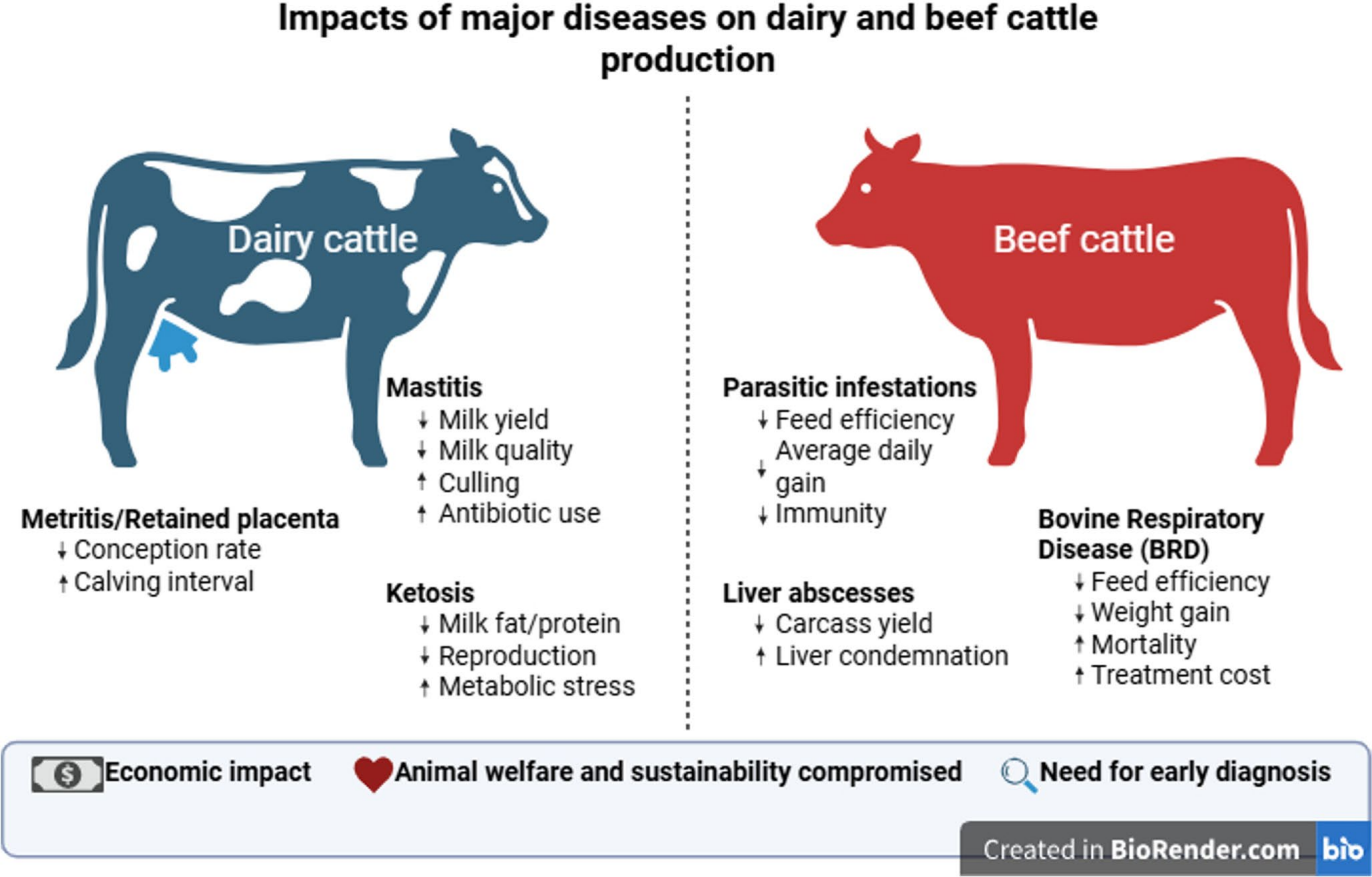
Impacts of major diseases on dairy and beef cattle production. Schematic representation of key production diseases and their effects on productivity, animal welfare, and sustainability in dairy and beef cattle. In dairy herds, mastitis, metritis/retained placenta, and ketosis are the primary causes of economic loss, reducing milk yield and quality while impairing fertility and metabolic stability. In beef operations, parasitic infestations, liver abscesses, and tick-borne diseases diminish feed efficiency, carcass yield, and growth performance. Collectively, these diseases compromise animal welfare, increase antibiotic use, and highlight the need for early and precise diagnostic strategies to enhance herd health and sustainability

Infectious, metabolic, reproductive, parasitic, and musculoskeletal disorders collectively impose major constraints on cattle productivity and sustainability, compromising both economic returns and animal welfare (Fig. 2). In dairy herds, delayed detection prolongs inflammation, reduces milk yield, and increases culling rates. At the global level, a Monte Carlo simulation across 183 countries estimated that 12 major dairy diseases generate approximately US$65 billion in annual global losses (standardized to 2024 values), with subclinical ketosis (US$18 billion), clinical mastitis (US$13 billion), and subclinical mastitis (US$9 billion) representing the largest contributors (Rasmussen et al. 2024). Beyond these high-burden infectious conditions, metabolic disorders, including hypocalcemia and fatty liver, are also prevalent during early lactation and are associated with reduced milk production and impaired reproductive performance (Wilkens et al. 2020; Melendez and Pinedo 2024). Similarly, uterine disorders such as metritis, retained placenta, and associated postpartum conditions are prevalent during early lactation and have been shown to delay conception and decrease reproductive efficiency.

In beef systems, economic losses are often less visible but equally substantial. Undiagnosed infections and stress-related immunosuppression reduce growth efficiency and carcass quality (Blakebrough-Hall et al. 2020). Bovine respiratory disease (BRD) remains the dominant feedlot health challenge, costing the U.S. beef industry over US$1 billion annually (O’Donoghue et al. 2025). Additional infectious and parasitic burdens, including lumpy skin disease, gastrointestinal nematodes, and cattle ticks, impair productivity through reduced fertility, weight gain, and increased organ condemnations (Strydom et al. 2023; Saqib et al. 2023).

A substantial proportion of economic losses in dairy production arises from delayed diagnosis and subclinical disease states that remain undetected under conventional monitoring systems. Given the high per-case costs associated with subclinical metabolic, inflammatory, and infectious disorders, even modest improvements in early detection efficiency may translate into meaningful herd-level savings.

Economic modeling approaches applied to automated lameness detection (ALD) systems provide a relevant illustration of this principle. Dynamic programming–based cost–benefit analyses estimated net returns ranging from $13 to $99 per cow per year, with the majority of simulated scenarios yielding positive long-term profitability over a 10-year horizon (Kaniyamattam et al. 2020). Although ALD systems rely on behavioral monitoring rather than biomarker analysis, the modeling framework illustrates how improved detection timing can generate measurable financial returns when coupled with timely intervention.

By analogy, biomarker–AI integration has the potential to reduce treatment costs, minimize involuntary culling, and preserve productive lactations. While the precise financial impact varies by management context, these projections suggest that incremental gains in predictive accuracy can yield scalable economic benefits across diverse production systems.

## The convergence of biomarkers, multi-omics, and AI in cattle health management

Traditional veterinary diagnostics have long relied on clinical examination, hematology, and microbiological culture. While these approaches remain essential components of herd health management, they are inherently reactive, often identifying disease only after clinical signs become evident. Limited sensitivity for subclinical conditions, dependence on laboratory infrastructure, and delayed turnaround times further constrain their utility for timely intervention and outbreak mitigation (Naveed 2025). In response to these limitations, biotechnology has emerged as a transformative driver of diagnostic innovation, enabling more rapid, precise, and field-applicable detection systems (Velayudhan and Naikare 2022; Miglio et al. 2023). As livestock production systems have intensified and economic margins narrowed, the need for earlier, more reliable, and scalable diagnostic strategies has grown substantially.

Within this evolving landscape, biotechnology has enabled the systematic identification and quantification of biomarkers as objective indicators of animal health. Biomarkers, measurable indicators of physiological or pathological processes, are increasingly recognized for their potential to enhance veterinary diagnostic strategies. Detectable in biological matrices such as blood, milk, urine, saliva, and feces, they encompass molecular, biochemical, and physiological parameters that reflect underlying health status (Perera et al. 2022). Depending on their intended use, biomarkers may serve diagnostic, prognostic, predictive, or monitoring purposes (Myers et al. 2017). Importantly, their principal advantage lies in their capacity to detect subclinical alterations prior to overt clinical manifestation, thereby facilitating a shift from reactive treatment toward proactive, prevention-oriented herd management (Eman et al. 2025).

Early biomarker research in cattle centered on inflammatory and metabolic indicators. Acute-phase proteins (APPs) such as haptoglobin and fibrinogen were established as systemic markers of inflammation, facilitating earlier recognition of mastitis and respiratory disease (McSherry et al. 1970; Eckersall and Conner 1988). Similarly, metabolic markers including β-hydroxybutyrate (BHB), non-esterified fatty acids (NEFA), and glucose became key indicators of negative energy balance in dairy cows (Adewuyi et al. 2005; Pedernera et al. 2008). Collectively, these advances marked a shift from purely clinical observation toward quantitative biological monitoring.

Subsequent advances in high-throughput technologies expanded biomarker research into genomic, transcriptomic, proteomic, and metabolomic domains. However, this expansion also introduced substantial analytical complexity. Multi-biomarker panels and integrated multi-omics datasets are characterized by nonlinear interactions, context-dependent effects, and multivariate dependencies that challenge conventional statistical modeling approaches (Perera et al. 2022). Consequently, defining robust and universally applicable diagnostic thresholds has become increasingly difficult. Although composite biomarker panels may enhance sensitivity and specificity, their clinical translation could benefit from analytical frameworks capable of effectively modeling high-dimensional biological relationships.

AI and machine learning (ML) methodologies provide the analytical capacity required to interpret high-dimensional, multivariate biomarker datasets (Javaid et al. 2025). Within Precision Livestock Farming (PLF) systems, these analytical approaches predominantly integrate production and behavioral variables to predict mastitis, lameness, and metabolic disturbances with moderate to high accuracy (Lasser et al. 2021; Zhou et al. 2022; Jiang et al. 2023). However, current AI-based health monitoring frameworks remain largely phenotypic, with molecular biomarkers still underutilized despite their biological relevance. This may be attributed, at least in part, to practical and methodological challenges, including cost, limited sampling frequency, assay standardization issues, small and heterogeneous omics datasets, and restricted cross-farm generalizability, as well as limited regulatory and clinical validation frameworks.

To advance toward true precision cattle health management, future approaches should incorporate genomic, transcriptomic, proteomic, and metabolomic data into biologically informed AI models capable of capturing systems-level interactions rather than isolated markers. Such integration has the potential to enhance diagnostic specificity, improve predictive robustness, and strengthen mechanistic interpretability (Wadood et al. 2025).

**Fig. 3:**
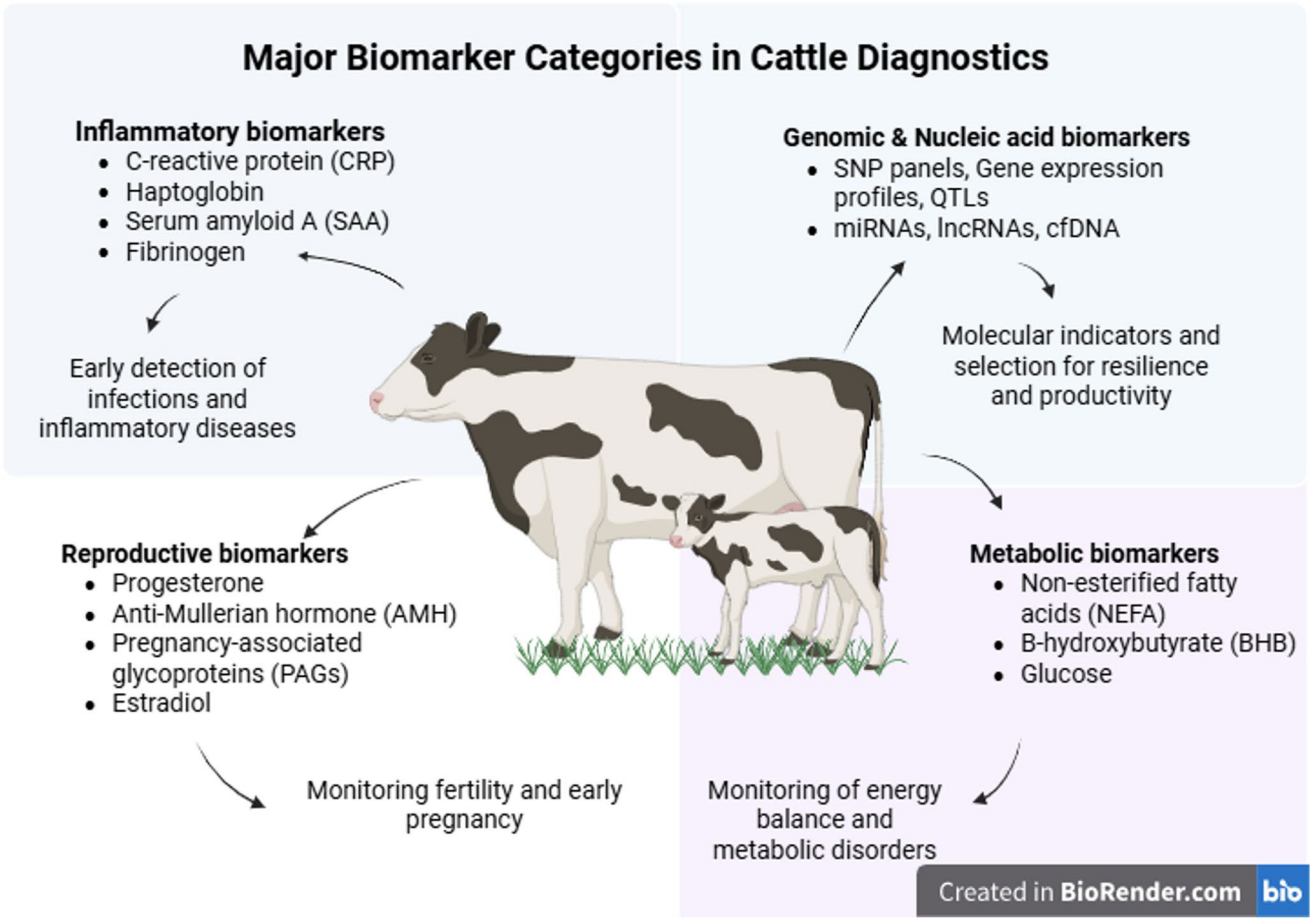
Overview of major biomarker categories used in cattle diagnostics

Accordingly, this review provides a structured synthesis of major bovine biomarkers, categorizing them into inflammatory, metabolic, reproductive, hormonal/stress-related, and genomic/molecular groups (Fig. 3). In the absence of a universally accepted classification framework, this structure is proposed to facilitate analytical coherence and comparative interpretation. Within each category, both the utility of biomarkers and the potential of AI-based analytical approaches are critically examined, emphasizing their integrative potential to advance precision diagnostics in cattle.

Inflammatory, reproductive, metabolic, and genomic/nucleic acid biomarkers provide complementary insights into cattle health and productivity. Inflammatory markers (e.g., Hp, SAA, CRP) support early detection of infections and inflammatory diseases, while reproductive biomarkers (e.g., progesterone, AMH, PAGs) are used to assess fertility potential and pregnancy status. Metabolic biomarkers (e.g., NEFA, BHB, glucose, and urea) allow monitoring of energy balance and metabolic disorders. Genomic and nucleic acid biomarkers (e.g., SNPs, miRNAs, cfDNA) serve as molecular indicators for disease resilience, productivity, and precision breeding.

### Inflammatory biomarkers in cattle health and disease diagnosis

Accurate and timely diagnosis of infectious diseases in cattle remains challenging due to their frequently subclinical onset, nonspecific clinical signs, and variability in host immune responses. In large herds, limited individual monitoring and overlapping disease manifestations further complicate early detection (Nadeem et al. 2025). Such delays often lead to empirical antimicrobial use, raising concerns regarding antimicrobial residues, resistance development, and long-term sustainability (Samtiya et al. 2022). These constraints underscore the need for sensitive, scalable diagnostic and early-warning systems.

Inflammatory biomarkers have therefore emerged as key tools for detecting immune activation prior to overt clinical disease. APPs, including C-reactive protein (CRP), haptoglobin, serum amyloid A (SAA), and fibrinogen, are induced by pro-inflammatory cytokines (IL-1β, IL-6, TNF-α) and increase during infectious and uterine disorders such as metritis and endometritis (Giagu et al. 2022; Saco and Bassols 2023). However, their diagnostic specificity is limited, as APP concentrations are influenced by physiological and environmental factors such as age, lactation stage, heat stress, transport, and analytical variability (Saco and Bassols 2023; Loch et al. 2023).

In parallel, SCC remains the primary milk-based indicator for mastitis; however, reported diagnostic performance varies considerably, with sensitivity ranging from 0.21 to 1.00, specificity from 0.21 to 0.95, and area under the ROC curve (AUC) values between 0.52 and 0.75 (Stanek et al. 2024). These findings indicate moderate and inconsistent discriminatory capacity, reflecting an inflammatory response rather than direct pathogen detection. Beyond mastitis, cytokine and chemokine signatures have shown diagnostic promise in chronic infections such as bovine tuberculosis, yet their application is limited by vaccination effects and biological variability, and requires further validation to establish robust diagnostic thresholds (Khalid et al. 2023). Collectively, while biologically informative, these markers lack disease specificity and are influenced by physiological and environmental confounders.

AI provides a framework to overcome these limitations by contextualizing inflammatory biomarkers within multidimensional data environments. ML models integrating SCC and milk electrical conductivity demonstrate high diagnostic performance in mastitis detection in experimental datasets, with neural network approaches achieving AUC values close to 0.98, reflecting the potential of advanced models to capture complex pattern relationships beyond traditional threshold-based methods (Pan et al. 2025). At a deeper molecular level, transcriptomic–AI integration has enabled multigene biosignature discovery, allowing classifiers to distinguish mastitic from healthy cows based on coordinated gene-expression networks rather than isolated biomarkers (Sharifi et al. 2018).

Beyond improving diagnostic accuracy, AI-driven integration enables several transformative advances. Temporal modeling facilitates the identification of preclinical trajectories, enabling prediction of disease risk before overt inflammatory elevation, while multimodal data fusion improves differentiation between infectious and non-infectious inflammatory states. In addition, probabilistic risk scoring supports targeted intervention strategies and optimized antimicrobial decision-making. At the herd level, adaptive AI systems enable continuous recalibration across breeds, management systems, environmental stressors, and seasonal variation, which is particularly relevant in multifactorial conditions. When supported by standardized measurement protocols and multi-farm validation, these approaches shift inflammatory diagnostics from static, threshold-based detection toward probabilistic, context-aware, and dynamically adaptive disease modeling, promoting antimicrobial stewardship and sustainable herd health management in alignment with One Health principles.

### Metabolic biomarkers in cattle health and production

Common metabolic disorders such as ketosis, hypocalcemia, hypomagnesemia, and fatty liver predominantly arise during the transition period (defined as the 3 weeks before and after parturition), when high milk production demands frequently exceed dietary energy intake, resulting in pronounced negative energy balance (NEB) (Lopreiato et al. 2020; Kang et al. 2025). These disorders may progress gradually and remain subclinical in their early stages, delaying intervention and amplifying downstream effects on reproductive performance, milk yield, and herd longevity (Cascone et al. 2022).

Metabolic biomarkers provide valuable insight into the physiological and nutritional status of dairy cattle. Circulating concentrations of NEFA and BHB are widely recognized indicators of lipid mobilization and ketone body production during NEB (Krnjaić et al. 2022). However, their diagnostic interpretation is influenced by physiological stage, management practices, sampling timing, and environmental stressors, limiting the generalizability of fixed cut-off values across herds (Stancheva and Penev 2025). For example, heat stress can independently elevate NEFA concentrations and activate inflammatory pathways, thereby mimicking NEB-associated metabolic signatures and complicating differential diagnosis (Stefanska et al. 2024).

These limitations highlight the constraints of static, single-time-point diagnostics in biologically dynamic systems and support the need for analytical frameworks capable of integrating longitudinal and multivariate data. AI-driven approaches, including machine learning (ML) models such as random forests, gradient boosting, and time-series algorithms, have been applied to capture nonlinear relationships among biomarkers, production traits, physiological indicators, and environmental variables (Grzesiak et al. 2025). By modeling temporal trajectories rather than isolated measurements, such approaches can improve early detection of metabolic disturbances by identifying deviations from individual baseline patterns.

Several studies illustrate this potential. For instance, milk Fourier-transform infrared (FTIR) spectroscopy combined with ML algorithms has demonstrated strong predictive performance for hyperketonemia by estimating BHB and NEFA concentrations from routinely collected milk spectra (Walleser et al. 2021), offering a scalable approach for herd-level monitoring (Martin et al. 2015). In addition, metabolomics–ML pipelines have identified early metabolic signatures associated with lameness during the precalving period, supporting links between systemic metabolic imbalance and locomotor disorders (Randall et al. 2023).

While most research has focused on energy-related biomarkers, mineral homeostasis represents another critical but often under-integrated component of metabolic regulation during the transition period, including disorders such as hypocalcemia and hypomagnesemia (Kang et al. 2025). Disruptions in calcium and magnesium homeostasis are associated with impaired neuromuscular function, metabolic instability, and increased disease susceptibility (Nithya et al. 2024; Grigė et al. 2025). An integrative approach combining blood biochemical markers with milk composition and sensor-derived behavioral data revealed that subclinical hypocalcemia is associated with coordinated disturbances in mineral, protein, and energy metabolism, even in the absence of clinical signs, highlighting the potential of multimodal data integration for early detection (Grigė et al. 2025).

Collectively, while conventional metabolic biomarkers remain indispensable in veterinary practice, their diagnostic value can be further enhanced when integrated within AI-based analytical frameworks. The transition from static threshold-based interpretation toward dynamic, herd-calibrated risk modeling may support earlier intervention, improved productivity, and more sustainable herd management.

### Reproductive and endocrine biomarkers

Reproductive biomarkers are central to fertility management, supporting monitoring of ovarian cyclicity, early pregnancy detection, and identification of embryonic loss or subclinical uterine disorders. Effective interpretation of these markers directly influences insemination timing, optimization of time to conception (days open), and overall herd reproductive efficiency.

Progesterone remains the most widely applied endocrine biomarker for assessing estrous cyclicity and pregnancy status (Madureira et al. 2021). Its utility depends strongly on the sampling matrix (milk versus blood) and the monitoring strategy employed, particularly whether measurements are obtained as single time points or through repeated sampling. Single-time-point progesterone measurements may misclassify cycle stage or fail to detect early luteal insufficiency due to pronounced temporal fluctuations and sensitivity to metabolic stress, heat stress, and individual variation in luteal dynamics, thereby limiting predictive precision. In contrast, serial progesterone profiling, especially through automated on-farm milk monitoring systems, is increasingly implemented in precision dairy systems to capture dynamic hormonal patterns and improve reproductive decision-making. Anti-Mullerian hormone (AMH), by contrast, reflects ovarian reserve and long-term reproductive potential rather than immediate fertility outcomes. While valuable for heifer selection and donor screening (Mossa and Ireland 2019), AMH does not capture dynamic changes in cycle stage, oocyte competence, or uterine inflammatory status, restricting its short-term clinical applicability.

Beyond endocrine markers, oxidative stress–related indicators such as malondialdehyde (MDA), superoxide dismutase (SOD), and glutathione peroxidase (GPx) have been associated with infertility, retained placenta, and endometritis (Dirandeh et al. 2021; Boni and Cecchini Gualandi 2022). Elevated NEFA and BHB concentrations further link metabolic imbalance to impaired follicular function and delayed ovulation, potentially compromising oocyte competence (Bruinjé and LeBlanc 2024). Although these markers provide mechanistic insight into the interaction between metabolism, inflammation, and reproductive physiology, their standalone diagnostic performance is limited by overlapping pathophysiological pathways and insufficient disease specificity. Consequently, fixed diagnostic thresholds often fail to capture early or multifactorial reproductive dysfunction.

Integrating endocrine, metabolic, and oxidative stress biomarkers offers a multidimensional representation of reproductive status (Mayasari et al. 2019), yet such composite panels introduce analytical complexity. Biomarkers often exhibit asynchronous temporal dynamics, differential biological weighting, and nonlinear interactions that challenge conventional statistical interpretation (Wadood et al. 2025). AI-based approaches may help address these limitations by modeling longitudinal biomarker trajectories, capturing multivariate dependencies, and recalibrating predictive probabilities across lactation stage and parity.

Building on this framework, AI-assisted reproductive modeling may extend beyond binary pregnancy detection toward probabilistic fertility prediction. By combining biomarker profiles with activity monitoring, milk yield variation, body condition scores, and environmental stress indicators, ML frameworks could generate individualized conception risk scores and help identify early deviations suggestive of embryonic loss or uterine dysfunction. Such dynamic risk stratification may enhance sensitivity to subtle reproductive disturbances while potentially reducing unnecessary interventions.

Rather than replacing established reproductive biomarkers, AI is positioned to augment their interpretability within adaptive, data-driven decision-support systems. This approach could support precision breeding strategies, optimize insemination timing, enable earlier intervention in high-risk cows, and ultimately improve reproductive efficiency at the herd level.

### Genomic and molecular biomarkers

#### Genomic biomarkers for disease susceptibility and health prediction

Genomic biomarkers are increasingly recognized as valuable tools for improving animal health by enabling early identification of inherited variants associated with disease susceptibility, immune competence, and physiological resilience in dairy cattle. Unlike conventional diagnostics that detect disease after clinical or biochemical changes occur, genomic information provides predictive, pre-clinical insight based on DNA variation present from birth, supporting early risk stratification and selective breeding strategies (Rojas de Oliveira et al. 2024; Kasimanickam et al. 2025).

Advances in livestock have facilitated the identification of immune-related genetic markers associated with resistance or susceptibility to infectious diseases (Rani et al. 2023). Polymorphisms in genes such as CD14, TLRs, CXCR1, and JAK2 have been linked to mastitis resistance and incorporated into genomic selection programs targeting improved immune resilience (Khan et al. 2023). Similarly, variants in innate immune genes including SLC11A1, NOD2, and MHC-II have been associated with differential susceptibility to Johne’s disease (Kravitz et al. 2021). These findings underscore the contribution of host genetics to disease risk and long-term herd robustness.

Although genomic selection programs have improved long-term herd resilience (Kasimanickam et al. 2025), the predictive gap between genetic risk and phenotypic disease expression under variable field conditions remains substantial. High heritability does not necessarily translate into robust real-time predictive performance, particularly under biologically heterogeneous field conditions. Consequently, genomic biomarkers are more informative for population-level risk stratification than for immediate clinical intervention.

Individual immune gene polymorphisms typically exert modest effect sizes and show population-dependent predictive value, limiting their utility as standalone diagnostic markers, as most traits require integration of many small-effect loci for reliable prediction (Tan et al. 2023). Moreover, genetic predisposition does not fully account for environmental, nutritional, and management-related factors that modulate phenotypic disease expression under field conditions (Silva Neto et al. 2024). Consequently, genomic biomarkers alone may be insufficient for real-time clinical decision-making.

AI may enhance the translational potential of genomic biomarkers by enabling polygenic risk modeling and integrative analysis across biological scales. Rather than assessing isolated variants, ML frameworks can aggregate thousands of SNP effects into composite genomic risk scores and model genotype–environment interactions that shape disease expression. The integration of genomic, transcriptomic, and production datasets further supports the identification of biologically coherent gene networks associated with immune resilience and metabolic stability. For instance, transcriptomic–ML strategies have successfully identified coordinated gene-expression signatures linked to complex production traits such as feed efficiency (Chen et al. 2021), underscoring the broader applicability of multi-omics AI frameworks in livestock systems.

Importantly, the predictive performance of AI-integrated genomic models varies substantially across traits and populations. For highly polygenic and low-heritability traits, improvements over conventional genomic evaluation methods are not always consistent, as machine learning approaches do not uniformly outperform traditional statistical models in genomic prediction (Chafai et al. 2023). In contrast, for complex traits shaped by genotype–environment interactions, nonlinear AI-based modeling may provide measurable gains in risk stratification. Therefore, the added predictive value of AI depends not only on algorithmic architecture but also on trait architecture, training population diversity, and data dimensionality.

In this context, genomic biomarkers may evolve from static breeding indicators into dynamic components of precision herd health systems when integrated within AI-driven predictive frameworks. Embedded within such models, genomic information could support herd-level disease-risk stratification and optimized breeding strategies aimed at enhancing genetic resilience. Over time, improved disease resistance at the population level may contribute to reduced treatment needs and more sustainable antimicrobial use, aligning genetic selection with long-term production and One Health objectives.

#### Nucleic acid–based biomarkers (miRNA, lncRNA, circRNA, cfDNA)

Among nucleic acid–based biomarkers, miRNAs are among the most extensively studied due to their conserved structure and central role in post-transcriptional gene regulation (Rani and Sengar 2022; Kappari et al. 2024). Dysregulated miRNA profiles have been associated with infectious and reproductive disorders, including mastitis and subfertility in dairy cows (Luoreng et al. 2021; Abeysinghe et al. 2025). Beyond miRNAs, other non-coding RNAs such as lncRNAs and circRNAs have been implicated in inflammatory, reproductive, and stress-related conditions, reflecting their broader involvement in immune and metabolic regulation (Sheybani et al. 2021; Hasankhani et al. 2023; Kirgiafini et al. 2024).

Despite their mechanistic relevance and high sensitivity, ncRNA biomarkers face significant translational challenges. Expression profiles are influenced by physiological state, breed, environmental stress, and sampling variability, and many reported associations remain correlative rather than causative (Oyelami et al. 2022). Moreover, heterogeneity in sequencing platforms, normalization strategies, and bioinformatic pipelines limits cross-study comparability and complicates the establishment of standardized diagnostic thresholds (Panahi et al. 2025). As a result, ncRNA signals, while biologically informative, require contextual interpretation within broader molecular and phenotypic frameworks.

Circulating cell-free DNA (cfDNA) and mitochondrial DNA (mtDNA) have increasingly been recognized as dynamic indicators of tissue injury, apoptosis, and systemic inflammation (Leishangthem et al. 2018; Goggs et al. 2020). Owing to its short half-life, cfDNA allows near real-time assessment of cellular turnover (Goggs et al. 2020), whereas mtDNA may function as a damage-associated molecular pattern (DAMP) that activates innate immune pathways (Zhou et al. 2024). Recent studies report elevated cfDNA levels in mastitis, heat stress, and enzootic bovine leukosis, highlighting its emerging role as a biomarker of inflammatory and stress-related conditions (Chen et al. 2023; Jahan et al. 2025). However, cfDNA interpretation remains constrained by limited tissue-of-origin resolution, rapid temporal variability, non-specific elevation across inflammatory states, and sensitivity to pre-analytical handling conditions (Aydın et al. 2025). Furthermore, limited external validation cohorts and inconsistent normalization strategies contribute to reproducibility challenges, underscoring the need for harmonized analytical pipelines before routine clinical deployment.

In this context, AI may provide an important analytical layer. AI-driven models can integrate ncRNA expression patterns, cfDNA dynamics, inflammatory markers, and production data to identify multivariate molecular signatures rather than relying on single-analyte thresholds. By capturing nonlinear interactions and temporal deviations across molecular networks, ML frameworks may enable biologically coherent disease-risk stratification at both individual and herd levels. Thus, the clinical utility of nucleic acid–based biomarkers may lie not only in isolated measurement, but increasingly in their integration within standardized, AI-supported, multi-omic diagnostic systems.

## Comparative insight: when do biomarkers truly benefit from AI?

A critical evaluation of the literature indicates that the diagnostic value of biomarkers in cattle health is highly context-dependent and strongly influenced by analytical methodology (Li et al. 2022; Heirbaut et al. 2023). Single biomarkers, particularly inflammatory and metabolic indicators, often demonstrate acceptable sensitivity but limited specificity when interpreted using fixed thresholds (Giagu et al. 2022; Bausewein et al. 2022; Heirbaut et al. 2023). This limitation becomes especially pronounced in multifactorial diseases such as mastitis, ketosis, and metritis, where overlapping physiological responses obscure disease-specific signals (Zhao et al. 2025).

In contrast, when multiple biomarkers are analyzed concurrently and interpreted within production, physiological, and environmental contexts, AI-based models may enhance diagnostic resolution by capturing nonlinear interactions and temporal dynamics that are not readily addressed by rule-based or linear methods. However, this advantage is not uniform and may depend on the biological complexity and structural properties of the biomarker system under consideration.

Biomarkers characterized by high variability, multidimensional regulatory networks, or dynamic temporal behavior, such as inflammatory, metabolic, and genomic markers, are likely to benefit more from AI-based integration. Conversely, biomarkers governed by relatively stable physiological thresholds, including certain endocrine indicators, may yield only limited incremental benefit from complex modeling approaches.

Taken together, these observations suggest that the value of AI lies not in universal superiority but in its capacity to resolve complexity where conventional interpretation reaches its limits. Table 2 synthesizes these comparative contexts and delineates where AI integration offers substantive diagnostic gain rather than incremental refinement.

**Table 2:** Comparative performance of biomarker-based diagnostics with and without AI integration in cattle

Biomarker category	Disease context	Conventional use (without AI)	AI-integrated approach	Added value of AI	Key limitations
Inflammatory (Hp, SAA, SCC)	Mastitis (subclinical/clinical)	Threshold-based detection; limited specificity	ML models integrating Hp, SAA, SCC, milk yield (Bobbo et al. 2021)	Earlier detection, improved sensitivity	Breed and lactation-stage variability
Metabolic (NEFA, BHB, glucose)	Ketosis, fatty liver	Single-marker cut-offs	ML models accounting for parity, DIM, and production history (Satoła and Bauer 2021; Taechachokevivat et al. 2024)	Risk prediction before clinical onset	Requires longitudinal data
Reproductive (Progesterone, AMH)	Estrus detection, fertility	Hormonal thresholds, manual interpretation	AI-driven temporal pattern analysis (Wang et al. 2022, 2025)	Improved insemination timing	Sensor and assay cost
Genomic (SNPs, CNV markers)	Disease susceptibility	Static genetic risk estimation	AI-enhanced genomic prediction models (Džermeikaitė et al. 2025)	Improved disease resilience selection	Population-specific bias
Nucleic acid (miRNA, cfDNA)	Mastitis, heat stress	Experimental, low clinical translation	AI-based multi-omics integration (Oyelami et al. 2022)	Captures early molecular perturbations	Limited field validation

The AI-integrated approaches and added values summarized in this table are conceptual projections based on available literature and related applications, as direct biomarker–AI integration studies in cattle remain limited

## Economic, sustainability, and one health implications

AI-enhanced biomarker diagnostics offer substantial economic and sustainability advantages in cattle production systems. Early detection of subclinical mastitis, ketosis, and inflammatory disorders preserves milk yield, reduces treatment costs, and minimizes premature culling, thereby improving herd profitability. By identifying at-risk animals before overt disease manifestation, these systems shift herd management from reactive treatment to proactive risk mitigation (Lasser et al. 2021).

From an antimicrobial resistance (AMR) perspective, AI-integrated biomarker frameworks reduce diagnostic uncertainty during early disease stages. Conventional herd management often relies on empirical antimicrobial administration in response to nonspecific clinical signs. By combining inflammatory, metabolic, and physiological biomarkers with AI-based pattern recognition, bacterial infectious processes requiring antimicrobial treatment can be distinguished from non-infectious metabolic or inflammatory disturbances, enabling evidence-based therapeutic decisions. This targeted, individual-animal approach reduces unnecessary antimicrobial exposure, limits selective pressure for resistant pathogens, and supports antimicrobial stewardship in alignment with One Health principles (Miranda 2024).

AI-driven diagnostic frameworks may improve reproductive outcomes by facilitating earlier identification of reproductive dysfunction and subclinical uterine or metabolic disturbances. When endocrine and metabolic biomarkers, such as progesterone, NEFA, and BHB, are analyzed longitudinally and integrated with contextual behavioral or sensor-derived data, ML models can detect deviations from normal cyclic or metabolic patterns before overt fertility failure becomes evident. Such integrative modeling approaches have the potential to refine ovulatory timing assessment and reproductive risk stratification compared with single-marker or observation-based strategies (Wang et al. 2020; Sakar et al. 2025). Earlier identification of reproductive instability may support timely intervention, with possible benefits for time to conception, pregnancy success, and overall herd reproductive stability. These improvements could contribute to reduced replacement pressure and enhanced productive lifespan, with downstream economic and sustainability implications.

Environmental benefits arise through improved feed efficiency and reduced disease-associated productivity losses. Healthier cows convert feed more efficiently into milk or meat, lowering greenhouse gas emissions per unit of output. Early disease control further decreases the environmental burden associated with prolonged illness and the rearing of replacement heifers, including feed consumption, water use, and methane emissions (Grandl et al. 2019; von Soosten et al. 2020).

Collectively, integrating biomarker data into AI-driven decision-support systems enables precise, individualized interventions that reduce unnecessary treatments, minimize drug residues and environmental contamination, and strengthen biosecurity and disease surveillance. Through its combined economic, ecological, and public health benefits, AI-enhanced biomarker diagnostics represent an important component of sustainable, precision-oriented livestock production.

## Challenges and future prospects

**Fig. 4:**
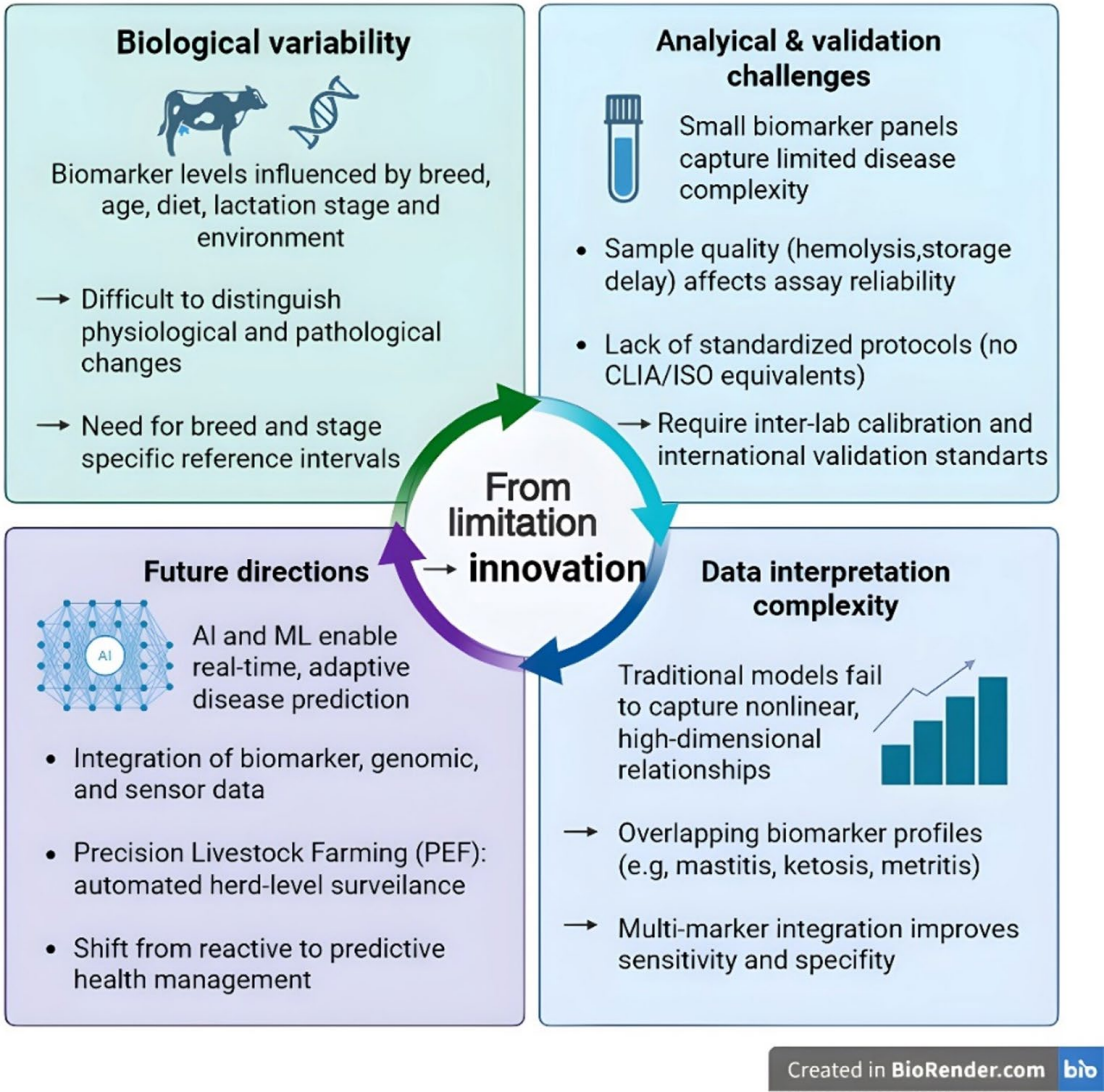
Conceptual framework linking biological variability, analytical limitations, and data-complexity challenges to AI-driven innovation in biomarker-based cattle diagnostics. Although promising results have been reported under controlled conditions, real-world implementation remains constrained by biological variability, analytical standardization gaps, data fragmentation, and field-level adoption barriers

Although AI-enhanced biomarker diagnostics represent a promising approach, several issues must be addressed to support their effective implementation in cattle health management (Fig. 4). A primary limitation concerns model transferability across breeds and regions. Most predictive models are trained on high-producing Holstein herds in Europe and North America, introducing breed- and region-specific bias that reduces generalizability to indigenous or crossbred cattle with distinct physiological and biomarker baselines (Mrode et al. 2019; Chafai et al. 2023). Differences in milk composition, metabolic profiles, and stress responses, for example between Holstein and Jersey cows (Lim et al. 2020; Joo et al. 2021), may lead to systematic misclassification when models are applied without recalibration. Similarly, indigenous and crossbred cattle often exhibit breed-specific immune and metabolic adaptations (Oke et al. 2025), further constraining model robustness. Addressing this bias requires coordinated multi-breed and multi-region datasets capable of generating representative biomarker baselines and enabling external validation prior to large-scale deployment. In parallel, transfer learning approaches, where models pre-trained on large Holstein datasets are fine-tuned using smaller, breed-specific cohorts, may offer a pragmatic strategy to adapt global AI frameworks to data-scarce indigenous populations.

Beyond biological variability, implementation is constrained by analytical and regulatory limitations that cannot be resolved by algorithmic refinement alone. Biomarker reliability is highly sensitive to pre-analytical factors, including sample handling and storage conditions, which compromise reproducibility (Rossi et al. 2021; Kovačević et al. 2024). Unlike human diagnostics governed by harmonized frameworks such as Clinical Laboratory Improvement Amendments (CLIA) and ISO 15,189 (Medical laboratories-Requirements for quality and competence), veterinary biomarker assays lack standardized international quality-control systems, and existing accreditation mechanisms remain limited in scope (Carter and Smith 2021). Furthermore, overlapping metabolic and inflammatory pathways in multifactorial disorders such as mastitis, ketosis, and metritis reduce disease specificity, limiting the reliability of single-marker diagnostics (Eman et al. 2025). To address these constraints, standardization should operate at three complementary levels: harmonized pre-analytical sampling and storage protocols to improve reproducibility; validated assays with breed- or production system–specific reference intervals to enhance biological interpretability; and minimum validation requirements for AI models, including external multi-farm testing, transparent performance reporting, and feature-attribution outputs (e.g., SHapley Additive exPlanations (SHAP), which provide interpretable measures of the contribution of each biomarker to the model output), prior to field deployment.

At the data infrastructure level, further challenges emerge from fragmented and inconsistently structured livestock health records. Robust AI performance depends on large, well-annotated, and interoperable datasets; however, limited cross-farm data sharing and the absence of centralized infrastructures restrict scalability and external validation, particularly in small-scale or extensive production systems. Models trained on narrow or single-farm datasets are therefore prone to overfitting, data imbalance, and reduced transferability (Distante et al. 2025). Strengthening data interoperability, promoting multi-farm training cohorts, and implementing transparent model-evaluation frameworks are thus essential to ensure robustness and generalizability.

From a field-level perspective, adoption barriers extend beyond algorithmic performance to include economic constraints, infrastructure demands, and human factors. Although AI offers transformative potential for livestock health, its uptake in real-world veterinary practice remains limited, particularly in rural and resource-constrained settings. High costs associated with sensors, computational infrastructure, and data storage pose significant challenges for small-scale farmers and underfunded clinics (Melak et al. 2024). In this context, SimHerd-based modeling of sensor-assisted herd management systems, although not specifically AI-driven, demonstrates that annual returns may range from −€33 to +€119 per cow, depending on herd conditions and management assumptions (Pfrombeck et al. 2025). This variability underscores that the economic viability of advanced digital technologies, including AI-enhanced diagnostics, is highly context-dependent and cannot be assumed solely based on technological sophistication.

Even when economically viable, AI systems may fail to achieve adoption if usability and interpretability are not adequately addressed. Effective integration requires not only financial feasibility but also technical literacy and clinician trust. Many veterinarians and farmers lack sufficient digital training to operate and interpret AI-based systems confidently (Ghavipanje et al. 2025). Model interpretability is therefore critical for clinical acceptance, as many deep learning models function as “black boxes.” Explainable artificial intelligence (XAI) encompasses a range of methodological approaches designed to enhance transparency by providing explicit feature attribution and decision pathways. Techniques such as SHAP and Local Interpretable Model-agnostic Explanations (LIME) allow visualization of the relative contribution of individual biomarkers to model predictions (Tjoa and Guan 2021; Saarela and Podgorelec 2024). In cattle health management, where AI outputs inform high-stakes decisions such as antimicrobial administration, reproductive interventions, and culling, transparent models are essential to ensure physiological plausibility, reinforce clinician trust, and enable responsible deployment (Neethirajan 2023). To bridge the gap between research and field implementation, future efforts should prioritize the development of explainable AI frameworks alongside edge computing solutions that support real-time, on-farm decision support.

Ethical and governance considerations warrant careful attention, as farm-level biomarker data are increasingly integrated into centralized AI platforms. Clear regulatory frameworks are essential to define data stewardship, usage rights, and informed consent, ensuring that scalability does not compromise farmer privacy. Beyond data ownership, ethical concerns extend to algorithmic fairness and equitable representation; models trained on imbalanced datasets may systematically misclassify under-represented breeds or low-input production systems (Mehrabi et al. 2022). Such biases introduce significant animal welfare risks, where false-positive predictions may lead to unnecessary antimicrobial use or premature culling, while false-negatives compromise timely clinical intervention. Ultimately, aligning AI systems with clinical reasoning requires interdisciplinary governance that protects data rights while ensuring that algorithmic outputs complement, rather than replace, professional veterinary judgment (Sezgin 2023).

Current biomarker research in cattle health has predominantly focused on milk and blood matrices, particularly in dairy systems and mastitis surveillance. However, growing attention has been directed toward alternative biological fluids, including urine, saliva, feces, and reproductive tract secretions, as complementary diagnostic sources (Perera et al. 2022). Recent multi-omics studies integrating metabolomic and microbiome data across multiple matrices have demonstrated that cross-compartment analyses provide a more comprehensive characterization of metabolic and microbial alterations than single-matrix approaches (Zhu et al. 2023; Li et al. 2025). From an AI perspective, such biological diversity represents an opportunity rather than a limitation. ML frameworks are well-suited to integrate heterogeneous, high-dimensional datasets and capture nonlinear relationships across biological compartments. As multi-omics and data-integration technologies advance, AI-driven models incorporating diverse matrices alongside conventional indicators may improve diagnostic specificity, enable earlier detection, and enhance the assessment of multifactorial cattle diseases.

While many previous reviews have examined biomarkers and AI separately, this synthesis evaluates their bidirectional interaction, emphasizing that AI performance is biologically contingent on standardized biomarker systems rather than algorithmic complexity alone. By linking biological variability, analytical standardization, and field-level implementation, this review advances a translational roadmap for precision cattle health. Moving forward, the priority must remain the development of explainable, fair, and cost-effective AI models supported by open data infrastructures. As computational capacity expands, AI is positioned to function as an adaptive decision-support partner, contributing to One Health objectives across diverse livestock systems. Ultimately, the clinical value of AI in veterinary medicine will depend not on its complexity, but on its seamless integration with biologically standardized and field-compatible systems.

## Conclusion

Biomarker-based diagnostics integrated with AI represent a critical advance in precision cattle health management. While single biomarkers and threshold-based approaches are limited by biological variability and low disease specificity, AI enables the integration of inflammatory, metabolic, reproductive, and molecular signals into predictive, context-aware diagnostic frameworks. The value of AI lies not in algorithmic sophistication alone, but in its capacity to interpret heterogeneous biomarker data alongside production and environmental variables. Despite encouraging performance, clinical translation remains constrained by limited standardization, population-specific bias, and insufficient field validation. Progress will require harmonized biomarker protocols, multi-breed datasets, interoperable data infrastructures, and XAI models aligned with veterinary decision-making processes. When biologically grounded and transparently validated, AI-enhanced biomarker systems may help improve early disease detection, reduce inappropriate antimicrobial use, and support sustainable, One Health–oriented cattle production. Ultimately, AI can meaningfully enhance precision diagnostics in cattle health only when multi-dimensional biomarker systems are standardized, interpretable, and embedded within clinically responsible analytical frameworks. Given the rapid evolution of biomarker and AI technologies, continuous evaluation and updating of these approaches will be essential to ensure their effective and sustainable implementation in cattle health management, necessitating ongoing critical reassessment of their use under real-world farm conditions.

## Data Availability

No datasets were generated or analysed during the current study.
